# Crossmodal counterpoint: from music to multimedia – incongruency, cognitive dissonance, irony, and surrealism

**DOI:** 10.3389/fpsyg.2026.1728329

**Published:** 2026-02-09

**Authors:** Charles Spence, Nicola Di Stefano

**Affiliations:** 1 Crossmodal Research Laboratory, Department of Experimental Psychology, University of Oxford, Oxford, United Kingdom; 2 Institute of Cognitive Sciences and Technologies, National Research Council, Rome, Italy

**Keywords:** (in)congruency, conflict, counterpoint, dissonance, multimedia, resonance, surrealism

## Abstract

Laboratory-based research on multisensory perception often presents participants with unpredictable combinations of auditory and visual stimuli that may be classed (by the experimenter) as either congruent or incongruent. Cognitive neuroscientists generally assume that congruent combinations of experimental stimuli will be processed more fluently and lead to enhanced crossmodal binding and multisensory integration than will incongruent combinations of stimuli. Typically, however, the participants involved in such laboratory research are given little if any information (or context) to explain why these seemingly random combinations of sensory stimuli are being presented. This situation differs markedly from the deliberate combination of eye and ear in an artistic context (say when music is added to film). In the latter case, conflict is sometimes introduced deliberately into a scene. A film director might, for example, choose to combine violent onscreen action with uplifting happy music. The presentation of such audiovisual emotional incongruency invites the viewer to reflect on what they are experiencing, and why this particular combination of stimuli has been chosen. Such crossmodal counterpoint (or cognitive dissonance) is sometimes used as a rhetorical device to introduce a note of irony. It is interesting to note how, in such cases, there is little sense of averaging the sensory inputs (or their emotional effects) as is so often seen when congruent inputs are presented in cognitive psychology studies. In this narrative historical review, we take a critical look at the concept of crossmodal counterpoint, and review the research and theorizing on its use.

## 1 Introduction

Typically, when music is added to film, it is congruent.^1^ In an artistic context, conflict (what might be described as crossmodal incongruency), is occasionally introduced deliberately into a scene (for its rhetorical distancing effect). A film director might, for example, choose to combine violent onscreen action with uplifting happy music (see Spence and Di Stefano, 2025c). The presentation of such obvious emotional incongruency between the eye and ear invites the viewer to reflect on what they are experiencing (Gorbman, 1980; Lipscomb and Tolchinsky, 2005), and why the director (artist, or multimedia content creator) may have chosen to pair this particular combination of stimuli (some commentators talk of this as a kind of ‘resonance’; Kargon, 2011; Lucier and Kane, 2016; Muecke and Zach, 2007). Such crossmodal counterpoint (or dissonance) may be used rhetorically to introduce a note of irony (Zabalbeascoa, 2003, 2008).^2^ According to Wingstedt et al. (2008, p. 195): “The rhetorical function refers to how music sometimes ‘steps forward’ to comment on the narrative events or situation. This is often achieved by having the musical expression contrast the visuals or by referring to well-known musical material.” Such a rhetorical function can perhaps be seen as a (musical) analogue to flashforward, offering a forward-looking commentary that contrasts with the retrospective nature of flashbacks.

Notice how, in such cases of audiovisual emotional dissonance, the perceiver does not naturally tend to average the sensory inputs or their emotional effects (Ernst and Banks, 2002), as so often occurs when pairs of sensory stimuli are presented in laboratory studies (e.g., Parke et al., 2007; see Spence and Di Stefano, 2025c, for a review).^3^ Rather, the intended ‘meaning’ of the audiovisual percept is only grasped if the two input streams are deliberately and intentionally kept separate.^4^ Crossmodal counterpoint, while often occurring at an emotional level, can also occur at more of a sensory, or structural level (e.g., Gunther and O’Modhrain, 2002).^5^ In this narrative historical review, we take a critical look at the concept (and deliberate use) of crossmodal emotional counterpoint in an artistic context and question the feasibility of providing insights for practitioners (e.g., film directors, and multisensory experience designers) that are based on laboratory research on multisensory perception from studies of the conflict situation. Before delving into the psychological literature, in the next section, we briefly examine the concept of counterpoint as originally formulated in the context of music.

## 2 Musical counterpoint

Counterpoint stands out as one of the most foundational compositional techniques in Western music. The term itself originates from the Latin *punctus contra punctum* and refers to the combination of two or more independent melodic lines that are played (or sung) simultaneously (Yust, 2018). While each of these lines is musically coherent on its own, when combined, they create a richer and more complex musical result. *The Oxford Dictionary of Music* concisely defines counterpoint as: “The ability, unique to music to say two things at once comprehensibly” (Kennedy, 2017, p. 198). This layered structure has intrigued composers for centuries, but it also offers fertile ground for psychological exploration.

Historically, counterpoint was developed and refined during the Renaissance and Baroque periods composers such as Palestrina, J. S. Bach, and later Mozart and Beethoven pushed the technique to its expressive and structural extremes (Hoag, 2018). While its roots are formal and rule-bound, good counterpoint often feels intuitive and emotionally resonant. This suggests a close alignment between musical structure and human cognitive preferences (see Spence and Di Stefano, 2022, on the notion of crossmodal harmony). Even those listeners lacking any formal musical training often respond positively to contrapuntal textures, indicating that our appreciation for musical independence and interdependence may be more innate than learned.

From a listener’s perspective, counterpoint poses a unique cognitive challenge, touching on several key themes: how we separate and integrate auditory streams (Bregman, 1990; Bregman and Rudnicky, 1975), how expectations are formed and fulfilled in music (Huron, 2006), and how complexity contributes to emotional and aesthetic experiences (e.g., Delplanque et al., 2019). In particular, the brain’s ability to perceive and enjoy multiple simultaneous melodies reflects its capacity for organizing overlapping information into coherent patterns. This aligns with principles from Gestalt psychology that emphasize the human tendency to seek meaningful wholes from diverse sensory inputs (Coppola, 2025; Wagemans, 2015). Much of the historical tradition of counterpoint is directed at creating multiple independent lines that interact seamlessly in order to create a unified, harmonious whole. J. S. Bach’s *Prelude and Fugue in C Major* from The Well-Tempered Clavier is a quintessential model of contrapuntal clarity. The fugue’s voices enter in orderly sequence, echoing a shared theme with rhythmic and harmonic precision.^6^ Similarly, Palestrina’s *Missa Papae Marcelli* exemplifies graceful counterpoint with vocal lines moving independently and smoothly blending into a coherent whole.

Musical counterpoint has been used as a powerful tool for evoking surprise through incongruity. Examples such as Ives’ *Unanswered Question* or Mozart’s sextet in *Don Giovanni* might illustrate how independent musical lines can be intentionally misaligned to provoke cognitive dissonance. In Ives’ piece, listeners are exposed to the superposition of different, contrasting layers that operate in separate tempos, keys, and emotional registers, never converging. The result is a disorienting and challenging auditory experience. Mozart’s sextet provides an example of how contrapuntal incongruity might be used in a different but equally compelling manner. In the sextet from Act II, six characters sing simultaneously, each expressing a distinct emotion and narrative perspective. The musical lines overlap and diverge, thereby creating a tapestry of conflicting motivations, perhaps mirroring the complexity of real-life social interactions. For the listener, this may create cognitive tension, as their attention shifts between characters, and coherence emerges only in hindsight (see also Davison and Banks, 2003; Palmer and Holleran, 1994; Sloboda and Edworthy, 1981; Taher et al., 2016).

Despite being a unisensory phenomenon, musical counterpoint may well represent a model for the broader concept of audiovisual counterpoint. It exemplifies how multiple elements can remain partially independent while nevertheless forming an intelligible whole, how alignment and misalignment can be used to generate tension or coherence, and how novel meanings can emerge from the interaction of components rather than from any element taken in isolation. These principles are not specific to music, but generalize to other domains in which distinct streams of information are combined. In what follows, we suggest that similar dynamics of independence, interdependence, and emergent structure play a central role in understanding crossmodal (audiovisual) relations in an artistic context.

## 3 Crossmodal counterpoint in film

Sound film, rather than silent film, emerged in the 1920s. Early film directors were both excited, and more than a little vexed, in thinking if, and how, to combine these two media. However, it is perhaps worth remembering that even in the era of silent film, there was often a live musical accompaniment, involving a pianist, organist, or even a small orchestra. Consider here, for example, only how the films shown at the 1900 Paris World Fair featured theatre actors performing sketches with synchronized gramophone accompaniment (Olsson, 1986; Wallis and Malm, 1988; see Spence and Di Stefano, n.d.).^7^ Notably, Charlie Chaplin, one of the era’s most iconic figures, often composed the musical scores for his own films, emphasizing the essential role music played in shaping the cinematic experience.

### 3.1 Crossmodal counterpoint in early film

The influential early Russian filmmaker, Sergei Eisenstein (1898–1948) talked of: “the creation of a new orchestral counterpoint of sight-images and sound-images” (Eisenstein et al., 1999). According to German film theorist and critic Siegfried Kracauer (1889–1962), counterpoint occurs when music and picture convey ‘different meanings’ that meet in a montage effect (Kracauer, 1960, p. 141): “Imagine the close-up of a sleeping face which appears to the rhythms of nightmarish music: it is all but inevitable that the intriguing discrepancy between these sounds and so peaceful a picture should puzzle us.”^8^ Eisenstein considered music to be one of the elements in the montage that comprises a film. As Buhler (2014, p. 190) notes: “Counterpoint in early sound film theory thus became synonymous with montage and with the asynchronous use of sound. The latter also becomes the privileged mode of sound, the sound that would not be, like synchronous sound, redundant with the image.”

When a viewer is presented with what may well appear to be seemingly random combinations of sensory stimuli, such arbitrary combinations of stimuli might even be considered Surrealist (see Gorbman, 1980; and Lynch, 1984, for Surrealism in the context of music videos).^9^ According to Gorbman (1980, p. 189): “whether a certain montage of elements is intended or not (surrealist word-games vs. traditional poetic activity, the drunken pianist vs. a score by John Williams), their corroboration will generate meanings. The point is that image, sound effects, dialogue, and music-track are absolutely inseparable during the viewing experience, and they form a *combinatoire* of expression.” (italics in original). The Frenchman Jean Cocteau (1889–1963) who was, amongst other things, a film director, supposedly scored some of his films on the principle of what he called ‘accidental synchronization’: that is, he would take George Auric’s music, carefully written for particular scenes in his film, and deliberately apply the ‘wrong’ music to the wrong scenes (Gorbman, 1980, p. 190).

Another famous early Russian filmmaker, Dziga Vertov (1896–1954),^10^ argued with Eisenstein, believing that before filming or shooting and editing, images and sounds could enter any kind of relationship. Nevertheless, as noted by Bulgakowa and Bordwell (2006), Vertov’s work in sound was actually based on the same principles of counterpoint and asynchrony as theorized by Eisenstein (see also Hubbert, 2008). In fact, on seeing Vertov’s work for the first time, the self-taught early composer of film music, Hanns Eisler (1898–1962; see Schweinhardt and Gall, 2014), stated that: “It is spectacular—the way the music attacks the image, the way the contradictions emerge between these two dimensions. This is all completely new, the most brilliant innovation that the sound film has delivered.” (cited in Bulgakowa and Bordwell, 2006, p. 224).

Vertov was particularly interested in the “associative potentials of sound and image, playing with the possibilities of their equivalence, and testing strategies of substitution by replacing image with sound and vice versa.” (Bulgakowa and Bordwell, 2006, pp. 226–227; and see Spence and Di Stefano, 2024, on the challenges of sensory translation). Vertov introduced an intriguing non-traditional, media-related switch in his 1931 film, *Enthusiasm*. The film starts by broadcasting the soundtrack, but the spectator sees what the girl-mediator is hearing, as if the circuits of perception have been connected incorrectly. The eye and the ear exchange places so that the ear ‘sees’. This switching of the auditory and the visual encapsulates “The Birth of the Radio-Ear as the Cine-Eye” as described by Vertov in his diaries (Bulgakowa and Bordwell, 2006, p. 228).^11^ As Bulgakowa and Bordwell (2006, p. 229) go on to note: “The sounds are imposed on the image like independent variables. They build an ironic, alienating, and analytical distance toward the images, while the camera imitates the movements of drunks and the subjective view of the praying people: it sways back and forth like an alcoholic and bows as if in prayer.” Notice how, in this case, it is the very independence of the sensory channels that leads to the ironic tone of the whole piece.

Writing almost half a century ago, Gorbman (1980, p. 189) was, though, critical of the limited range of alternatives put forward by those considering the possible relations between the auditory and visual channel: “The restricted number of possible film/music relationships as discussed by most scholars seems curiously primitive, limited largely to the concepts of *parallelism* and *counterpoint*. Either the music “resembles” or it “contradicts” the action or mood of what happens on the screen.”^12^ Gorbman (1980, p. 190) goes on to ask: “Is there no other way to qualify film music which does not lie between these opposites but outside them? If we must summarize music/diegesis relationships in two words or less, the notion of *mutual implication* might help us at least to consider the problem better, and with the respect due to films of any complexity. For it is debatable that information conveyed by disparate media can justifiably be called *the same* or *different*.” The last point directly refers to the issue of similarity across the senses. In fact, as highlighted by Di Stefano and Spence (2023), the notion of perceptual similarity is inherently problematic when applied to sensory information conveyed by different senses. This is because different sensory modalities lack shared physical dimensions, making any kind of direct comparisons difficult. Similarities might be based on various cognitive factors, including shared emotional meanings, learned associations, abstract analogies, or structural alignments (isomorphisms) rather than true perceptual resemblance. This thus makes it difficult to clarify the meaning of the idea of “mutual implications” evoked by Gorbman in the preceding quote.

The unlikely existence of crossmodal perceptual similarity, and the lack of clear criteria to ground music/film association, may result in a sceptical attitude, namely, the idea that any music could accompany a given segment of a film. As Gorbman (1980, p. 190) put it: “In fact, whatever music is applied to a film segment will *do something*, will have an effect-just as whatever two words a poet puts together will produce a meaning different from that of each word separately.” This apparently provocative claim means that no pairing of music and film is inherently wrong. It can only be ineffective relative to the specific function or effect that the director intends to achieve. Audiovisual matching in film is, then, not simply a matter of sensory translation between audition and vision (Spence and Di Stefano, 2024), since there is no objectively correct match that would serve as a metaphorical equivalent of a ‘correct translation’. As Gorbman (1980, p. 190) notes: “If instead of orchestrated folk music a sudden tense dissonance or Indian drumbeat were to “hit” the characters in *Stagecoach* as they wend their way across Monument Valley, we would drastically revise our mental inventory of interpretations of the drama of the moment. To demonstrate the interdependence of music and film diegesis, we might use the tool of commutation by taking any small segment of film and applying different types of music to it. The *Stagecoach* example already suggests the dramatic importance of tension-producing harmonies and pauses, as well as general style.”

### 3.2 Crossmodal counterpoint in contemporary film

In the context of film music, crossmodal ‘dramaturgical counterpoint’ (what Schweinhardt and Gall, 2014, describe as the most famous and colourful term in Adorno and Eisler’s, 1946/1994, *Composing for the Films*)^13^ is most often observed when the emotional tone of the background music fails to match with the emotional tone of the on-screen action (Spence, 2020; Zhuang, 2023; see Spence and Di Stefano, 2025c, for a review). Scholars of film history have drawn attention to numerous examples of the use of emotional crossmodal counterpoint. Dümling (1998) points to Eisler’s music for Resnais’ short video documentary film *Night and Fog* (1955). He suggests that it represents musical counterpoint in the context of the cinematic portrayal of the unimaginable terror associated with the Holocaust. So, for example, Eisler composed deliberately unsentimental music for the gruesome documentary shots of gas chambers and piles of corpses, while a tragic melody for string orchestra accompanies the beautiful opening colour shots of the countryside (that the viewer subsequently realizes are shot from within an overgrown concentration camp). This use of dramaturgical counterpoint (where the music alludes particularly to what is not explicitly *shown*) is also linked to Adorno and Eisler’s (1946/1994) book (Eisler, 1947): “in which both authors declared their break with the aesthetics of the Hollywood motion picture industry and pleaded for a more significant and autonomous role for music in film.” (Dümling, 1998, p. 578).^14^ According to Dümling (1998, p. 581), the composer sought to “create a sense of detachment from the overwhelming power of horror”. As Resnais noted: “The more horrible the scenes, the more friendly the music. Eisler wanted to show that human optimism and hope could even exists in a concentration camp.”

Crossmodal emotional counterpoint appears once again in a particularly violent rape scene in the 1972 film version of Anthony Burgess’s (1962) dystopian satirical black comedy *A Clockwork Orange* (Burgess, 1962) is accompanied by ‘Singin’ in the Rain’ (Lipscomb and Tolchinsky, 2005; cf. Audissino, 2017). Opening in the same year, the baptismal ceremony with solemn organ music in *The Godfather* (1972), is juxtaposed with the brutal murders of rivals, highlighting Michael Corleone’s transformation into a ruthless mafia boss, and the hypocrisy of his double life. In the 1991 movie, *The Silence of the Lambs*, directed by Jonathan Demme, Bach’s “Aria” from the Goldberg Variations plays while Hannibal Lecter brutally killed two guards while executing his prison escape; This results in the contrast between the ethereal grace of Bach’s music and the violence unfolding on screen (Cenciarelli, 2012).^15^ Meanwhile, an intense shootout is accompanied by ‘Somewhere Over the Rainbow’ in the 1997 movie *Face/Off*.

Crossmodal counterpoint is also used in a scene from the 1987 film *The Secret of My Success*; There, the main character, Brantley Foster is about to be seduced by his boss’s wife in a swimming pool. The wife’s removal of Brantley’s swimming trunks is accompanied by the *Jaws* leitmotiv. As Wingstedt et al. (2008, pp. 205-206) note: “One possible reading of the scene is that the contrast between the playfulness of the situation and the serious and dramatic music creates a humorous effect. The use of the Jaws leitmotif here relies on the assumption that the audience is also familiar with the movie Jaws and its distinctive leitmotiv. The intertextual associations make us compare Vera to the ruthless shark, which can be seen as contributing humour to the scene (one of several available readings). On the interpersonal level, the music is here given a commenting role, producing a distancing perspective. It is almost as if someone was looking into the camera giving the audience a knowing and ironic wink.” Notice again how we see mention of the use of incongruency to create distance and an ironic theme. Ireland (2012, 2015, 2017) has also written extensively on the way that classical and popular music are frequently paired with extreme violence in contemporary movies (e.g., those of *mélomane* directors such as Quentin Tarantino; see Garner, 2001; Coulthard, 2009, p. 5). For instance, in *The Hateful Eight* (2015), David Hess’s delicate piano and acoustic guitar song ‘Now You’re All Alone’ is played while one character hunts down another by following a trail of blood through the snow before executing him. Ireland (2017) suggests that the use of dramaturgic counterpoint in this case can be considered as anempathetic (Chion, 1994, see §3.4 below).

### 3.3 How do contemporary audiences respond to audiovisual counterpoint in film?

According to Lipscomb and Tolchinsky (2005, p. 397): “Such a mismatch can invite intellectual processing and active participation (Lipscomb and Kendall, 1994). The audience member asks – consciously or unconsciously – what is the intended meaning? How do I resolve the conflict between the incompatible meanings I am receiving from the sound and image? If the music is familiar, the audience member may ask: How does this music I am used to hearing in one context relate to what am seeing now?” Such thoughts undoubtedly reflect a higher level of intellectualizing (and not one that will necessarily be triggered automatically given the absence of any obvious conflict; e.g., as in the case of mood music). Strachan (2006, pp. 195-196) similarly highlights the cognitive challenge that is likely to be associated with appreciating the ironic distance elicited by the use of crossmodal counterpoint in the music video genre (see also Allan, 1990; Aufderheide, 1986; Straw, 1993; Switchenberg, 1992). As Strachan (2006, p. 196) puts it: “A successful decoding of the text, however, requires a highly developed level of media literacy, and with it, an implied critique of the means and strategies of the media industries.”

### 3.4 Crossmodal counterpoint and Chion’s anempathetic function

According to Wingstedt et al. (2008, p. 207), diegetic music is sometimes used with what Chion (1994) calls an *anempathetic* function. That is, being ‘indifferent’ to the dramatic situation, thus resulting in a contrasting or commenting role (see also Sonnenschein, 2001). Audissino (2017) draws attention to the fact that in some cases of crossmodal emotional counterpoint, the relevant piece of music may well start prior to the scene with which it has been intentionally paired, and continue after the scene has finished. In the attempted gang-bang rape scene set in an abandoned theatre in Stanley Kubrick’s (1971) *A Clockwork Orange*, a hideous act of violence is shown on screen (as per the novel on which the film is based). However, the crossmodal emotional counterpoint is provided by Rossini’s carefree sounding overture from the comic opera *The Thieving Magpie* (1817).

According to Audissino (2017, pp. 81-82): “The ‘anempathetic effect’ happens in those instances in which some diegetic sound (a sound whose source is within the narrative world) is playing before a dramatic event and continues playing after it, unaffected by what happened. This produces in a scene an unsettling sense of ‘cosmic indifference’ (Chion, 1994: 8–9): the ‘uncaring’ sound is taken to signify that individual sufferance is completely irrelevant to the economy of the Universe, we are helplessly alone. Though formulated to account for the agency of diegetic sound and music, non-diegetic music too is often singled out as responsible for the anempathetic effect. It is in these terms that Sonnenschein applies Chion’s concept to the scene in?: [the viewer’s] involvement can be heightened when there is a great tragedy or catastrophe depicted, using the juxtaposition of happy music that simply challenges us to identify more closely with the victims, as in *A Clockwork Orange* (Sonnenschein, 2001, p. 156).” Here, note, music is treated as a rather mechanical modifier, something that changes the polarity of the visuals and perhaps also results in the viewer identifying with the victim. If music of opposite emotional sign is paired with a tragic event, then an anempathetic effect is likely to be the result and as a consequence we cannot help but pity the helpless victim.

### 3.5 Interim summary

While a large number of examples of crossmodal emotional counterpoint have been identified in the context of contemporary film, it is important to recognize that such examples are themselves quite rare (at least when set against the full range of situations in which film music is used). At the same time, the use of crossmodal emotional counterpoint tends to be short-lasting (e.g., for a scene, say).^16^ In this sense crossmodal emotional counterpoint is unlike some of the famous historical examples of musical counterpoint that were mentioned earlier, where, for example, the counterpoint runs through Bach’s *Goldberg* Var*iations* (i.e., in musical counterpoint there is a temporally extended relationship between the parts). It is perhaps relevant to note here that in the laboratory context, Gau and Noppeney (2016) have demonstrated that participants are more likely to bind mismatching auditory and visual speech (in the McGurk effect) if the McGurk stimuli trials are embedded within a stream of congruent auditory and visual speech tokens, rather than within a block of incongruent speech sounds and lip-movements. In this sense, one could perhaps talk of a ‘perceptual set’ to integrate auditory and visual inputs (cf. Epstein and Rock, 1960; Liu, 1976). Such a suggestion feeds into the literature on crossmodal Gestalt perceptual grouping (see Spence, 2015; Spence and Di Stefano, 2025a, 2025b). The viewer’s response to crossmodal conflict (in the psychology lab), or to the use of crossmodal emotional counterpoint in the context of film music is, in other words, likely to be determined, at least in part, by the particular context in which it is experienced.

## 4 Laboratory research on crossmodal congruency/counterpoint

This is not the place to review the many studies that have examined the consequences for perception, interpretation, and memory of the pairing of film scenes with music that is either congruent, neutral (or absent), or incongruent with the emotional content of the depicted scene (see Spence and Di Stefano, 2025c, for a review of that literature). Nevertheless, there are a few studies that are perhaps worth describing for their broader relation to the art of filmmaking, or interesting responses to crossmodal conflict that were elicited. In one study, for example, Hansen and Krygowski (1994) measured physiological arousal and the priming effects of sexually themed music video on people’s interpretation of a subsequently-presented ‘ambiguous’ television commercial. Participants’ interpretations of the latter were shown to be modified after viewing rock music videos. In particular, they were more likely to identify sexual connotations in the test commercial. Furthermore, increasing people’s level of arousal by having them cycle vigorously on a stationary bike led to still more extreme responses to the commercial. These findings were taken to support an arousal route to the impact of, say, music on the perception of film.

Hung (2000) explored music’s contribution to meaning-making in the context of television advertising, focusing on the effects of the (in-)congruency between musical and visual elements. Using a mixed-method approach, viewers’ interpretations of a couple of carefully chosen coffee commercials (created by pairing different music tracks and videos), were analyzed. Commercials were typically considered congruent by the participants when the music and visuals came from the same original ad, and incongruent when they had been intentionally mismatched. The congruent pairings appeared to reinforce coherent cultural narratives (e.g., of a natural or sophisticated coffee), while some incongruent combinations can also produce meaningful interpretations when viewers are able to draw on familiar cultural frames (e.g., action film tropes). However, other mismatched pairings led to confusion and negative evaluations of the product depicted instead. As such, Hung’s results challenge the simplistic notion of music as merely serving as an emotional cue, extending its functions as a cultural symbol whose impact depends on its relationship to other ad components and the viewer’s interpretive context.

Baranowski and Hecht (2017b) reported that the emotional tone of music that was paired with neutral scenes (happy music, no music, or sad music), significantly affected participants’ judgments of facial expressions (themselves happy, neutral, or sad) that were intercut with the scenes.^17^ Such examples demonstrate how a scene/image with neutral or ambiguous emotion can be pulled toward the more extreme emotion that is presented in the background music. Crossmodal effects such as these can perhaps fruitfully be framed in terms of Smith's (1999) ‘polarisation’ and ‘affective congruence’ account of the emotional contribution of film music. Polarisation refers to an interaction in which the specific affective character of the music moves the content of the picture toward the emotional pole communicated by the music. By contrast, affective congruence refers to those situations in which the viewer matches the affective components of the score to the emotional shading of narrative. According to Smith (1999, p. 148): “More than the sum of its parts, affective congruence produces a degree of emotional engagement that is stronger than either that produced by the music or visual track alone.” Notice here how the language appears to be hinting at a kind of emotional superadditivity (see Spence, 2025; see also Audissino, 2017).

Damjanovic and Kawalec (2021) studied the role of music-induced emotions on people’s recognition memory of filmed events. These researchers investigated the effects of pairing a comedic movie trailer (for the film *Table 19*) with emotive music on subsequent recognition memory of the events depicted in the trailer. In an independent groups design, the comedic trailer was paired with happy music (congruent condition) or sad music (incongruent condition). A no music condition served as the control. Participants in the incongruent condition displayed a recognition memory advantage for visual test items over participants in the congruent and control conditions. While changes in self-reported positive and negative affect did not correlate significantly with recognition memory, the perception of emotion-specific categories did. These findings therefore help to establish an empirical basis of ironic contrast techniques. They also hint at an affective component in the integration and representation of audiovisual action that is likely to emerge where a participant perceives or recognizes expressed emotions in music, without necessarily feeling an overall positive or negative affect.

In terms of the present review, what is particularly striking about the 25 or so published studies in this area (again, see Spence and Di Stefano, 2025c, for a recent review of this large body of research), is how the participants are typically not given any information concerning the relation between the stimuli (music and film clips). What is more, the participants in certain of the studies have been bombarded by a whole sequence of seemingly randomly combined auditory and visual clips in rapid succession (up to as many as 32 in Bolivar et al.’s, 1994, study; see also Hoeckner et al., 2011).

## 5 On the varied relations between auditory and visual information/stimuli in audiovisuals

### 5.1 On the multiple functions of musical narrative

**Figure 1:**
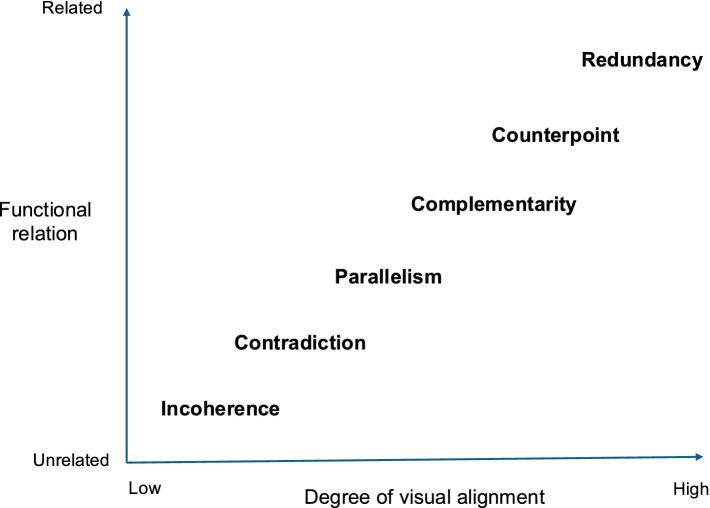
Schematic representation of the different types of audiovisual relationship arranged in terms of the ‘degree of audiovisual alignment’ and the ‘functional relation’ between the auditory and visual elements.

According to Wingstedt et al. (2008), musical narrative (e.g., as it appears in film and other multimedia) may have one of several functions. These include: The Emotive; The Informative; The Descriptive; The Guiding; The Temporal; and The Rhetorical (mentioned briefly earlier). Elsewhere, Zabalbeascoa (2008) categorizes the relationship between auditory and visual streams as: Complementarity (when the various elements are interpreted interdependently, i.e., they depend on each other for a full grasp of their meaning potential and function); Redundancy (repetitions (total or partial) that are regarded as unnecessary, superfluous or dispensable) – this what Sally Banes, in the context of the olfactory augmentation of live performance, refers to as pleonastic (i.e., pointless repetition; Banes, 2001); Contradiction ((or incongruity): defeated expectations of some sort of surprising combination to create such effects as irony, paradox, parody, satire, humour, metaphor, symbolism); Incoherence (inability to combine elements meaningfully); Separability (when unisensory message works better – as when soundtrack becomes a successful audio recording); and Aesthetic quality (author’s intention to produce something of beauty by a certain combination of elements). See Figure 1 for a schematic presentation or the various outcomes that may be observed as a function of the differing degree of audiovisual alignment considering the functional alignment of the stimuli.

### 5.2 ‘Resonance’, superadditivity, and sensory dominance

While the focus in this article is squarely on what auditory counterpoint does to the interpretation of visual stimuli, things undoubtedly operate in the other direction too. As Wingstedt et al. (2008, p. 194) note: “Just as the music will affect how we see things, the visuals will also determine how we hear the music. Murch (in Chion, 1994) describes a phenomenon he calls conceptual resonance between image and sound, where the sound makes us see the image differently, and then this new image makes us hear the sound differently, which in turn makes us see something else in the image and so on.” One might also consider linking the notion of ‘resonance’ with that of ‘superadditivity’. The latter phenomenon occurs when the combined effect of two or more unimodal stimuli is greater than the sum of their individual parts (Stein and Meredith, 1993).

Wingstedt et al. (2008. p. 193) go on to comment on the: “focus on the intermodal relationships of music and image. The examples illustrate how musical and visual expressions combine to form multimodal statements where the whole is certainly different than the sum of the parts….” Later in the same article, the researchers continue: “As the above examples show, there is in narrative multimedia more to see than meets the eye. When image, dialogue, sound effects and music combine into multimodal texts, a ‘chemical reaction’ can sometimes take place. The resulting whole is, if maybe not greater, certainly different than the sum of the parts. The communicational act takes place on several levels and through many simultaneous channels or modes, but our experience is perceived as being one. Since such experiences often are interpreted as being of primarily visual nature, the effect is, as stated initially, that what (we think) we see is to a large extent determined by what we hear” (Wingstedt et al., 2008, p. 208).

In fact, one could argue that, when incongruence arises between auditory and visual elements, the observer typically perceives the musical background as misplaced relative to the visual scene, which establishes the normative frame of reference. However, if the audiovisual pairing were truly symmetrical, one might just as plausibly conclude that, in the mentioned scene from *The Silence of the Lambs*, it is Bach’s music that is appropriate, while the actions shown onscreen depicted fail to cohere with it.^18^ Yet this reversal of normative priority seems intuitively odd.^19^ This asymmetry likely reflects the fact that films are perceived as primarily visual objects: The moving image tends to anchor perception, providing the dominant framework within which other sensory modalities, including sound, are interpreted and evaluated (cf. Percheron and Butzel, 1980). As Wingstedt et al. (2008, p. 194) note: “As audience however, our conscious attention is usually on the visuals. We tend to interpret the events on film or television as something we see – even if we in fact actually ‘hear/see’ it. This is reflected also in how we talk about media experiences: we go to see a movie, we watch television and so on. Language, in this case, may reflect the historical development of cinema, which originated primarily as a visual medium. For the sake of analysis however, emphasis will in the following primarily be put on music’s contribution to how meaning is established in the multimodal interplay of the filmic narrative.” (see also Hutmacher, 2019).

In her early commentary, Gorbman (1980, p. 190) highlighted how the notions of parallel and counterpoint erroneously assume the image as autonomous: “The very examples used by Kracauer show how music helps the viewer to *define* the images, themselves polysemic. Eisler comments on the inadequacy of the notion of parallelism: “From the aesthetic point of view, this relation is not one of similarity, but, as a rule, one of question and answer, affirmation and negation, appearance and essence. This is dictated by the divergence of the media in question and the specific nature of each.” (Eisler, 1947, p. 70).” Along somewhat similar lines, Strachan (2006, pp. 201-202) points to the fact that: “Just as the musical text is polysemic (i.e., its “meaning” is not fixed, it can have several signifieds, and its understanding may relate to the listener’s *a priori* knowledge, age, race, gender, etc.), so the video can be seen to work in the same way” ….[certain music videos] “can be understood simply as audiovisual texts in which the visual images complement the music and the visual editing follows rhythmic and structural elements of the song. On another, they utilize a complex layered set of visual signifiers that serve to contextualize and reflect the music in very specific ways.”

### 5.3 More complex organizational structure of audiovisual media

Over-and-above the organizational relations of individual auditory and visual items in the context of film, it is also possible to consider the rhythm of multimedia texts themselves. For example, Martinec (2000) distinguishes between mono-synchrony and poly-synchrony as two fundamental modes of temporal coordination across semiotic systems (here music and film). Mono-synchrony occurs when multiple modalities—such as gesture, speech, music, or movement—are closely aligned at all, or nearly all, rhythmic levels, creating a unified temporal structure. This form of synchronization is, for instance, typically found in the tight rhythmic alignment of voice and instrumental accompaniment in much popular music. In such cases, the perception of the different modes fuses into a coherent temporal Gestalt. In contrast, poly-synchrony arises when the semiotic modes are not fully synchronized across rhythmic levels. Poly-synchrony characterizes many multimodal performances, such as stage acting or film, where bodily movement, spoken dialogue, and background music follow overlapping but distinct temporal trajectories. Martinec highlights how differences in temporal organization across the auditory and visual channels can influence whether multimodal communication is experienced as a unified whole or as a layered, partially autonomous interplay of sensory streams.

However, while Martinec’s (2000) distinction between mono-synchrony and poly-synchrony offers a useful initial taxonomy, it also raises some important questions about the underlying notion of synchronization itself. Synchronization must be defined relative to a particular frame of reference. One could ask: Synchronized with respect to what? Temporal coordination could be evaluated against the beat structure of a musical score, the internal rhythm of speech production, the kinetic pacing of bodily movement, or even the broader perceptual expectations of an observer. Different modalities may exhibit local synchronizations without aligning globally, or may appear synchronized at one temporal scale (e.g., overall tempo), but asynchronous at another (e.g., fine-grained rhythmic subdivisions, see Spence and Di Stefano, 2025a, for a recent review).

Furthermore, what counts as ‘true’ synchronization may vary depending on the cultural context, the perceptual sensitivities of the audience, and the attentional distribution across sensory modalities. Thus, any assertion of mono- or poly-synchrony must implicitly select a privileged temporal structure as the normative basis for assessing coordination. Obviously, recognizing this relativity complicates simple categorizations and suggests that multimodal temporal organization is not merely a matter of objective timing but also of interpretive framing and perceptual/emotional attunement. At the same time, however, it should also be noted how different film genres may have different norms. As Björnberg (1994) has pointed out, the tropes and techniques common in the music video format are better understood in relation to the syntactic characteristics of popular music. That is, they are traits and editing techniques that attempt to visually echo and reflect the rhythmic, structural, and melodic properties of a given musical text (cf. Rabinowitz, 2004).

### 5.4 Comparing musical vs. audiovisual counterpoint

In music, the combination of independent melodic lines is guided by well-established compositional rules that define what constitutes effective or stylistically appropriate relationships—particularly in traditions like Baroque counterpoint, where dissonance, voice leading, and harmonic structure follow strict conventions. A composer can assess how a student has carried out the compositional task using counterpoint, identifying specific errors or rule violations with respect to the traditional framework. By contrast, the pairing of music with film visuals operates with far fewer formalized rules. That is, there is no universally accepted system that dictates what kind of musical accompaniment “fits” a particular scene, and it would be hard to imagine there are errors in such artistic practice. Effectiveness is instead evaluated in terms of narrative coherence, emotional resonance, genre conventions, and directorial intent.^20^

As a result, film music allows for a much wider spectrum of interpretive freedom. A musical cue may underscore, contrast, or even subvert the visual content, and still be considered successful if it serves a deliberate expressive function. However, this lack of fixed rules should not be taken as implying randomness, but rather points to a more context-sensitive form of coherence. In this sense, audiovisual matching resembles a looser, more fluid kind of counterpoint—one in which contrast can perhaps be just as meaningful as consonance. Whereas traditional counterpoint in music is bound by stylistic norms, filmic crossmodal counterpoint is open to ambiguity, irony, and disruption. The “rightness” of a pairing lies not in technical correctness, but in its perceptual and emotional impact on the viewer.

Returning here finally to the famous early German film music composer, Hanns Eisler, it is worth quoting Schweinhardt and Gall (2014, p. 173) at length concerning the latter’s key demand of film music, namely that: “The composer’s task was to impart the true perspective of the scene to the spectator” (*CftF*, 28). If we understand this as a further prescriptive premise together with the notion of musical illustration of the film action discussed above (that is, the demand for illustration that does not *merely double* but *interprets*), then the “true perspective” in Adorno and Eisler’s concept of dramaturgical counterpoint becomes clear—and, following from this, so also is revealed the sum, essence, and vision of Eisler’s work for film. It is in no way the case that music is limited to permanently contradicting the image or text levels of a film, which would then live up to neither the specific semiotic possibilities of these levels nor even a solid understanding of the term “counterpoint”; instead, film music *qua music* should achieve its dramaturgical sovereignty in a “collective of independent arts” (in the spirit of Brecht) and thus become an indispensable and independent element of the filmic narrative. This requires planning of the content and structure across all levels of the film from the beginning, that is, competent musical planning as early as the script-writing stage (preferably in cooperation with the composer). Only in this way may film music fulfil its key task, that is, to be “essential to the meaning of [a] scene” (*CftF*, 24).”

### 5.5 Intentional incongruency: crossmodal counterpoint as a rhetorical device

It would seem plausible to assume that the viewer of a film clip would respond rather differently if they thought that a seemingly-incongruent piece of music had been chosen deliberately by a film-maker to conflict in terms of its emotional valence with the visual scene rather than merely reflecting an arbitrary pairing. That said, we are not aware of anyone who has addressed this issue experimentally as yet (though see Fujiyama et al., 2012). As Zabalbeascoa (2008, p. 24) notes: “the music in a film may be original or not, but what matters most, from a textual and communicative point of view, is the relationship established between the music, and the script, and the photography, and how they all add up and combine with each other, so that viewers can interpret them in a certain way.” Other commentators who have addressed the theme of multimodal semiotics include Cook (1998) and Kress and Van Leeuwen (2006).

One of the most intriguing aspects of the use of counterpoint in film, therefore, is its power to provoke thought through incongruency (see also Maille and Fleck, 2011). When watching a movie, viewers typically expect that music will match the emotional tone of a scene—tense strings for suspense, soft piano for romance, say.^21^ While audiovisual congruency facilitates interpretation and enhances narrative fluency (Parke et al., 2007; cf. Csikszentmihalyi, 1990), when that harmony is disrupted—and when what we hear does not align emotionally with what we see—cognitive tension arises It is at this point that viewers are likely to start questioning what they see, being pushed to engage more deeply with the unfolding meaning. Thus, incongruent music in film does more than just “not fit”; it may trigger questions and interpretation. In contrast, congruent music simply accompanies/supports the visuals.

The effect of combining emotionally incongruent sound and image creates aesthetic/surface dissonance that demands deeper resolution—not in the narrative, but in the viewer’s mind (Spence and Di Stefano, 2025c). For instance, the juxtaposition of violence and musical grace in the scene from *The Silence of the Lambs* (mentioned earlier) is shocking, precisely because it breaks the assumed audiovisual practice (or convention). This violation of expectation triggers a kind of interpretive reflex: We search for a different narrative or symbolic meaning, often perceiving irony, surrealism, or psychological depth to account for such an incongruent match. At the same time, of course,monitoring multiple channels requires additional attentional resources.

Crossmodal counterpoint can be thus conceived of as a conceptual/rhetoric device that creates a tension between the two sensory modalities, ‘dislodging’ (or interrupting) passive viewing and requiring active cognitive engagement. Importantly, incongruent pairings tend to stand out precisely because they are infrequent and short-lived, typically embedded in a larger context of audiovisual harmony. In this way, counterpoint does not just disrupt; it shifts attention, reveals hidden layers, and compels us to reflect on how meaning emerges from the complex interplay of sensory inputs. In film—and in multimedia more broadly—counterpoint operates not merely as a stylistic flourish (or rhetorical filmic device), but as a mechanism for aesthetic inquiry and possibly as a means of provoking some sort of conceptual resonance (cf. Lucier and Kane, 2016; Manjarrez et al., 2007).

This heightened engagement may be partly explained by the inverted-U model of aesthetic response (Berlyne, 1971), according to which moderate levels of novelty or incongruity tend to maximize interest and attention. When music in film closely aligns with the visual and narrative tone, it may foster ease of processing (or flow; Csikszentmihalyi, 1990) while at the same time resulting in more or less passive viewing. At the other extreme, if the incongruity is too extreme, or else appears arbitrary—such as music perceived as unintentionally mismatched—it may alienate the viewer or disrupt meaning-making altogether. However, well-calibrated counterpoint, where the mismatch is clearly purposeful, or emotionally charged, can produce an optimal zone of interpretive engagement. Viewers are nudged to resolve or make sense of a mild cognitive dissonance, resulting in deeper reflection and a more layered aesthetic experience. This may help to explain why emotionally incongruent music often leaves a stronger impression and can shift interpretation in ways that are subtle or radical.^22^ The perceived intentionality behind such mismatching also plays a key role. As noted, it matters whether viewers believe the incongruence reflects an expressive directorial choice or instead a random experimental manipulation. These nuances highlight how counterpoint in the context of film music operates not only as a stylistic device but as a form of cognitive and emotional modulation. It serves to carefully balance fluency and disruption in order to guide the viewer’s attention and hence the meaning that the latter makes of the scene.

Finally, here, it should be recognized how the viewer may not necessarily always have a clear understanding of who is actually responsible for the introduction of crossmodal emotional counterpoint to film. As Wingstedt et al. (2008, p. 201) note: “Equally complex and hazy is usually the viewer’s idea about who is the producer(s) of the musical score in a film. Seldom is the musical under-score distinguished from the other aspects of the movie, making the issue of determining the musical communicator(s) vague and unreflected – just as the music itself is often experienced on an unconscious and unreflected level, de-emphasizing the listener’s awareness of any specific ‘musical communicator’. The implied author might be – more or less consciously – associated with different participants (one specific or a combination of several), such as the film director, the movie company or television network, the executive producer, the writer, the composer, the performing actors, even characters of the narrative or the rather indistinct notion of ‘the film itself’.” Of course, matters may become all the more complex when a novel is turned into a film. While, in such cases, it is likely to be the film director who has chosen to introduce any crossmodal counterpoint (as in the rape scene from *A Clockwork Orange*), that might not always be the case (as in the explicit mention of Beethoven’s 9th Symphony in Burgess’s original novel, or of Bach in Harris’s, 2002, *The Silence of the Lambs*).

## 6 Crossmodal counterpoint and crossmodal gestalt grouping

Film studies has been approached both from the direction of Gestalt psychology, but also from the qualitatively distinct approach of cognitive psychology. One of the important points to have emerged from this narrative historical review has been to highlight how laboratory-based studies of film music (or for that matter, multisensory perception) fail to capture the real-world experience of film. More than a decade ago, Donnelly (2014, pp. 18-24) made much the same point when highlighting that: “Although I am more than happy to accept the insights provided by cognitive psychology […] there are distinct aspects of the aesthetic process (for film especially) that are poorly accounted for by such an approach. […] The grasping of situations as a whole is one of the most profound insights of Gestalt psychology. […] Human hardware is determinedly pattern-seeking, looking for – and inevitably finding – some sort of sense, be it narrative, representational, relational, or whatever.”^23^ These results are consistent with Shevy’s (2013, p. 72) claim that: “Media effects do not occur in a vacuum; situational, cultural, social, and historical factors may alter the way in which users perceive, interpret, and otherwise respond to musical media. *Context* refers to the environment, literally and metaphorically, in which media effects occur.” Film, then, should not be considered as an imitation of life (Arnheim, 1932; Brooks, 1984; Cutting, 2005; Tan, 2018).

So how exactly should we think about the relationship between crossmodal counterpoint and Gestalt perceptual grouping? When resolved by the viewer, does the audiovisually incongruent stimuli result in anything that could be classed as ‘perceptual coherence’ (Handel, 2006)? Not ‘grouping by similarity’ as such, but rather some form of ‘grouping by contrast’ or common timing (i.e., common fate; cf. Spence et al., 2007; or what Cohen, 2013, refers to as congruency – though see below)? The following distinction between the cognitivist and Gestalt approach to film studies has been highlighted by D’Aloia (2025, p. 376): “Whereas the New Look’s theory of visual perception adopted by cognitivist film scholar (Bordwell, 1989, p. 18) posits that perception is influenced by cognitive factors such as expectations, experiences, and motivations, and emphasizes top-down processing (where higher-level cognitive processes influence lower-level ones), Gestalt privileges the bottom-up approach and focuses on how we naturally organize visual information into whole forms using innate principles.”

Potentially relevant here, returning to the musical roots of counterpoint, *The Harvard Dictionary of Music* (pp. 216–219) defines musical counterpoint as: “The combination of two or more melodic lines” requires complex demands on listeners’ attending to the tension between changes taking place in the horizontal (temporal) and vertical dimensions (think chord units) at the same time by observing “the perception of these relationships simultaneously is the perception of counterpoint.” (Randel, 2003, p. 216). This description sounds pretty close to the Gestalt perspective (see also Audissino, 2017; Meyer, 1956, p. 31). Consider only how Ehrenfels referred to ‘gestalt quality’ as the relational structure amongst elements that transcends the elements themselves (Ehrenfels, 1937; Ehrenfels and Smith, 1988).^24^ The word ‘gestalt’ refers to a ‘form/shape’ but more accurately to a dynamic process of organisation and a relation amongst the parts of a system. It is better rendered with ‘configuration’. The main focus of Gestalt is to study why we experience phenomena as wholes even if they are made up of separate components (see Ehrenfels, 1937; Köhler, 1970). The key question perhaps then comes down to whether or not music plus visuals should be treated as one whole, or as two disparate parts, especially in those cases of emotional counterpoint.

Audissino (2017) has argued against the ‘separatist conception’ of music and film, which the author attributes to an inveterate visual bias in Film Studies. According to Audissino (2017): “Films, even after the coming of sound, have typically been considered to be a pre-eminently visual medium, with the audio part being an addendum of secondary importance. An offspring of this conception is the polarisation of the role of music between parallelism (music replicates what is in the visuals) and counterpoint (music contradicts what is in the visuals), both positions implying that the visual element is the dominant, while music can merely be either subservient to it or a contestant. More recent theorisations still show the trace of the visual bias, for example, Carroll’s notion of music as a ‘modifier’, which again implies that the visual is the dominant element and music cannot but modify it (Carroll, 1996). Audissino (2017) argue that visuals and music should not be considered as two separate and unequal elements that are somehow pasted to each other but as two equal agents that fuse to create the audiovisual experience. The holistic view of Gestalt can be a good solution to overcome the ‘separatist conception’. There is, however, probably going to be no easy answer to this philosophical question, given that Spence and Bayne (2015) have argued at length against the existence of multisensory consciousness (see also Spence and Di Stefano, 2026).

The perceptual foundations of musical counterpoint align closely with the principles of Gestalt psychology. Concepts such as figure-ground segregation, similarity, proximity, and good continuation are central to how listeners perceive and follow independent melodic lines in polyphonic music. Gestalt theory emphasizes the brain’s tendency to organize sensory input into coherent wholes, and in the context of counterpoint, this means that multiple melodic voices are heard as distinct yet interrelated entities. The cognitive ability to both segregate and integrate simultaneous streams is essential for the perception of counterpoint and has been studied extensively in the field of auditory scene analysis (Bregman, 1990). Terhardt (1987) presented the Gestalt concept of Hierarchical Processing of Categories (HPC) and applied it to music perception, discussing musical tones, chords, and melodies in the light of the HPC concept. Meanwhile, while Huron (2001) draws on perceptual principles to explain voice-leading practices (see also Cohen and Dubnov, 1996).

Audissino (2017) proposes a Gestalt Psychology-based method for film music analysis, emphasizing the holistic fusion of sound and image in film. Gestalt principles offer a better framework for understanding how music and visuals create a synergistic audiovisual experience—not just visuals ‘modified’ by music. He introduces a method called ‘micro/macro configuration analysis’, which analyzes how the secondary parameters of music (e.g., tempo, register, timbre) align or contrast with visual elements (e.g., editing, mise-en-scène) to form a unified experience (macro-configuration). Using examples from *Citizen Kane*, *Hook*, *The Hateful Eight*, and *A Clockwork Orange*, Audissino shows how musical and visual elements can either mirror each other (parallelism), add new meaning (complementation), or even clash (counterpoint). However, they always as co-equal agents, advocating a view where music and visuals are fused into a single expressive system.

## 7 On the problematic notion of audiovisual (in-)congruency

**Figure 2:**
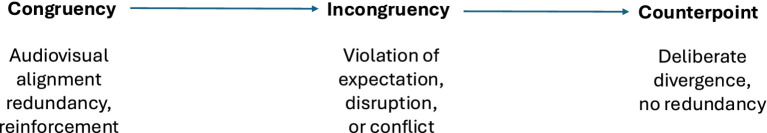
Schematic representation of the path from congruency, through incongruency, to counterpoint.

Let us return, in closing, to the problematic notion of crossmodal congruency. As noted already, this term would appear to be used in different ways even by those working withing the same field of study, never mind by those working in different disciplines (such as film studies, cognitive psychology, Gestalt psychology, etc.; Ireland, 2015). Cohen, for example, relates ‘congruency’ in her influential ‘Congruency-Association Model’ (e.g., Cohen, 2013), to the structural similarity (i.e., the synchrony of the auditory and visual inputs), though, as was mentioned earlier, congruency cannot be linked to perceptual similarity across the senses (Di Stefano and Spence, 2023). By contrast, in the field of cognitive psychology, congruency typically refers to the literature on semantic or crossmodal correspondences (Chen and Spence, 2010; Spence, 2011). Ireland (2015) highlights the problem thus: “‘Congruence’ and ‘incongruence’ are often operationally defined in relation to particular dimensions of the audiovisual relationship that researchers wish to measure. This creates specificity which is characteristic of the empirical approach and aids the potential replication of studies. Yet, this approach can be reductive and may emphasize different attributes dependent on the aspects of the film-music relationship that happens to be studied: Structural or temporal congruence may connote fit whilst semantic or mood congruence may more readily imply notions of appropriateness. The terminological specificity afforded by experimental designs must be explicitly explained and contextualized to ensure conceptual clarity.” Meanwhile, in the field of film studies, congruency is typically related to the emotional tone of the auditory and visual clips that are presented together (Bolivar et al., 1994; Boltz, 2004; see Spence & Di Stefano, n.d., for a review). Ireland (2017) highlights how incongruities can be perceived on various structural, semantic, or holistic levels in an audiovisual relationship (Heiser, 2016; Vandaele, 2002) (see Figure 2).

However, over-and-above such problems of definition, there are also problems of the reception of the deliberate use of incongruency in the context of film (Mera, 2002). For instance, informal testing by Ireland (2015) has highlighted the fact that the members of a film club whom he interviewed, often failed to mention the dramaturgical use of auditory–visual conflict in the short commercial film clips that he selected to show them. Use of the term congruency is also problematic when it comes to cultural stereotyping. Buhler (2014) brings up the example of the use of furious drumming to signify the unseen presence of ‘savage; Indians in many western movies. Congruency here is presumably defined by prior co-occurrence, such that the music becomes some kind of unfortunate pernicious *leitmotiv* for the audience who happens to be familiar with the trope (Buhler, 2014). As the film music composer Tiomin (1894–1979) notes, following exposure to such regular association, the actual sound of Indian song (what would presumably actually be more semantically congruent with the characters who are being depicted) does not have anything like the same emotional effect on cinema audiences. Tiomkin (1951, pp. 21-22) writes: “For instance, all audiences think a certain type of steady beat of tom-tom or tympani drum, and a high, wailing wind instrument performing in a simple four or five-tone scale, connotes one thing: Indians…If while the white settlers are resting or enjoying themselves, the background music suddenly takes on that tympani beat, the effect on the audience is electrifying. All know the Redmen are on the warpath even before the camera pans to the smoke signals on a distant hilltop. If I introduced genuine, absolutely authentic Indian tribal music, it probably would not have any effect at all.”^25^ Tiomkin refers to this as a “conditioned reflex.”

Finally, as has been mentioned already, it would appear that several possible different interpretations may be associated with the use of dramaturgic counterpoint (or incongruence). Which effect or interpretation, if any, a given viewer will come away (distancing, irony, analogy, polarization or contrast) with depends on a number of factors including their general media awareness, their prior cultural experience of film, as well as other possibly stylistic factors associated with director (Ireland, 2017), and/or the genre of film is which it appears (e.g., Björnberg, 1994). Given such considerations, it would seem highly unlikely that the perception of crossmodal counterpoint, particularly emotional incongruence between the music and the visual scene, is culturally universal. The perceived attributes of musical structures—such as the negative connotation of dissonance and the positive of consonance—are likely shaped through cultural learning and exposure (e.g., see McDermott et al., 2016). Similarly, the interpretation of visual emotional cues, including facial expressions, bodily gestures, and narrative pacing, varies considerably as a function of culture. As a result, what counts as incongruent in the pairing of soundtrack and imagery may also differ according to culturally specific norms of emotional communication and musical meaning. Thus, crossmodal counterpoint should be understood not simply as an objective feature of audiovisual structure but as a perceptual phenomenon mediated by cultural frameworks of meaning, expectation, and interpretation. As such, what is potentially needed is what David Ireland calls a ‘psycho-semiotic approach’, one that is contextualized by poststructuralist thought (see Figure 2). Future experimental research could investigate cross-cultural differences in the detection and interpretation of audiovisual emotional incongruence, shedding light on how cultural learning shapes crossmodal counterpoint and, more in general, multisensory integration processes.

## 8 Conclusion

Taken together, the evidence reported in this narrative historical review clearly supports that the notion of counterpoint (albeit defined as an exclusively auditory phenomenon by some commentators; Kennedy, 2017) can be extended beyond the unisensory auditory or musical case where it first originated. Unless the conditions are such that it is clear to the viewer/participant that an audiovisual mismatch is intentional, crossmodal incongruency may be all that is perceived. Under such conditions a range of outcomes may result, including distancing from the events portrayed in the story, irony, cognitive dissonance, resonance, or even surrealism. In the context of film, crossmodal counterpoint is, then, a rhetorical filmic device It is sometimes used to introduce a note of irony. The intentionality, the rhetorical device use of incongruency is not a feature of the situation that is necessarily captured by the laboratory research, especially that conducted in cognitive psychology (Ireland, 2015; Willemsen and Kiss, 2015; see Spence and Di Stefano, 2025c, for a review) (see Table 1 for a summary of some of the key themes, challenges associated with laboratory research, and considerations relevant to the artistic use of crossmodal counterpoint).

**Table 1:** Summary of some of the key themes relevant to the study of audiovisual (in)congruency, challenges associated with laboratory research on crossmodal interactions, and some considerations relevant to the artistic use of crossmodal counterpoint.

Key themes	Laboratory multisensory research	Artistic crossmodal counterpoint
Stimulus combination	Unpredictable pairings	Intentional, designed pairings
Context given to participant	Minimal or absent context	Rich narrative or thematic context
Goal of participant’s task	Measure integration efficiency (binding, fluency)	Create cognitive tension, irony, resonance
Typical outcome	Averaging, sensory fusion	Emotional dissonance, reflective engagement
Perceptual organization	Unified Gestalt via congruency	Parallel streams or deliberate tension
Interpretive effort	Minimal (automatic binding)	High (active cognitive interpretation)
Temporal structure	Short stimulus presentations	Extended scenes embedded in narrative arcs

## Footnotes

1 As Gorbman (1980) notes, audiences nowadays expect to hear sound and music while watching a film.

2 Anticipations of these techniques can be found in certain operas. There, music and dramatic action sometimes deliberately operate in ironic or dissonant relation. For instance, in *Don Giovanni*, Mozart pairs the upbeat Champagne Aria with the titular character’s predatory scheming, masking menace with levity. Or think about Verdi’s *Rigoletto* where the carefree “La donna è mobile” is deliberately juxtaposed with the tragic unravelling of the plot, creating sharp dramatic irony.

3 In fact, measures are often taken by the experimenters to avoid the participants in their laboratory studies from becoming aware of any perceptual conflict between the stimuli they happen to be presented with (see Rock and Victor, 1964, for one such early example).

4 Here, one might think in terms of the literature on the ‘disconfirmation of expectations’ (Schifferstein, 2001; Yeomans et al., 2008). However, note that in such cases, one stimulus obviously comes before another and is thus able to set an observer’s expectations; This is simply not the case in film where the music, congruent or otherwise, co-occurs with the unfolding of the visual scene. Perhaps a better comparison is with the literature on assimilation vs. contrast (Hovland et al., 1957; Piqueras-Fiszman and Spence, 2015; Zellner et al., 2004).

5 Crossmodal counterpoint can presumably operate at multiple levels from the sensory (Gunther and O’Modhrain, 2002) through to the emotional (e.g., Gorbman, 1980). Gunther and O’Modhrain (2002, p. 375) briefly note how: “a composer might wish to confound the observer’s sense of space, presenting conflicting auditory and tactile cues; essentially a *cross-modal counterpoint*.” (italics in original) This suggestion appears in the context of a consideration of how the stimulation of a vibrating bodysuit could be combined artistically with simultaneously presented music. Notice how, in this case, the crossmodal counterpoint would appear to be predicated at the structural level (thus more immediately aligning it with Gestalt grouping principles; see Baranowski and Hecht, 2017a; O’Leary and Rhodes, 1984; Spence, 2015; Spence et al., 2007). That said, the majority of the research that has been published to date would appear to have been devoted to studying the impact of what can best be described as audiovisual emotional counterpoint.

6 The Canadian pianist and writer, Glenn Gould, was famously fascinated by Bach’s contrapunctal or polyphonic music (Bartine, 2019).

7 See Hernández Barbosa (2015, 2023) for the live musical accompaniment that serenaded the public lucky enough to enjoy the immersive multisensory experience that was the *Maréorama.* This experience was also offered to the general public at the 1900 World Fair.

8 Kracauer’s reactions to a drunken movie-house pianist from his childhood, whose inattention to the screen resulted in pleasingly unorthodox audio-visual combinations, recall the Surrealists’ delight in discovery on every plane of life where there issued a “fortuitous encounter” (see Kracauer, 1960, p. 14).

9 Notice how the potential combination of stimuli do not themselves uniquely identify a particular example as Surrealist, rather that depends on the intention behind their co-occurrence.

10 The pseudonym was a play on the Ukrainian words for “spinning top”; His actual name was David Abelevich Kaufman.

11 It seems clear that in the early days of sound film, especially in Russia, it was an open playing field as far as the way in which auditory and visual stimuli might relate to each other (Hansen, 1999). There was also interest in the translating, or switching of the senses, as if to ‘see sound’ or ‘hear film’.

12 This pair of alternative relations can be traced back to the debate surrounding the novelty of sound cinema in the late 1920s (Wierzbicki, 2009, pp. 96–101). Spottiswoode (1950) referred to situations where the source of the sound lay outside the image as ‘contrapuntal’ (cf. Kargon, 2011).

13 That said, as Schweinhardt and Gall (2014, p. 173) note, the topic is dealt with surprisingly briefly in *Composing for the Films.* There, one finds the following: “The following examples show how music, instead of limiting itself to conventional reinforcement of the action or mood, can throw its meaning into relief by setting itself in opposition to what is being shown on the screen” (26)—is followed by three more examples from Eisler films: the model “Movement as a Contrast to Rest” is demonstrated with a sequence from *Kuhle Wampe* in which the “passive” mood of the images is contrasted with sharp, brisk, and dynamic music. The opposite situation, “Rest as a Contrast to Movement” is illustrated with a scene from *Dans les rues* in which a bloody fight is depicted with “tender, sad, rather remote” music (*CftF*, 27). Finally, the authors give an outline of the musical structure of a short scene in *Hangmen Also Die!* where the dying Reinhard Heydrich lies in his hospital bed: “The music consists of brilliant, strident, almost elegant sequences, in a very high register, suggesting the German colloquial phrase *auf dem letzten Lock pfeifend* (literally, ‘to blow through the last hole’, which corresponds to the English: ‘To be on one’s last legs’). The accompaniment figure is synchronized with the associative motive of the scene: the dripping of the blood is marked by a pizzicato in the strings and a piano figure in a high register” (*CftF*, 28).”

14 Renais wrote of working with Eisler that: “Above all, he showed me how to avoid musical redundancies…he nevertheless showed me how to apply music to create something akin to a ‘second level of perception,’ something additional, contrariwise. For example, one could simplify the music the most during points of high drama and, vice versa, elaborate it significantly at moments when the eyes are no longer engaged. In this way, an equilibrium may be reached wherein the viewer finds a balance between both seeing and hearing.” (quoted in Dümling, 1998, pp. 578–579).

15 The choice of music for this example of emotional crossmodal counterpoint perhaps not coincidental, given that Bach’s Goldberg Variations (Cenciarelli, 2012, p. 109) are one of the quintessential examples of musical (structural) counterpoint.

16 The one exception to this generalization being Resnais’ short video documentary film *Night and Fog* (1955). There, one finds multiple examples of deliberately dramaturgical counterpoint in Hanns Eisler’s music (Dümling, 1998).

17 The authors describe this as an auditory version of the Kuleshov effect, after the early Russian filmmaker Lev Kuleshov (1899–1970).

18 Perhaps worth noting here, the reference to Bach appears in Thomas Harris’s original novel (see Harris, 2002), as well as in the four following films (Cenciarelli, 2012).

19 Similarly, according to Buhler (2014, p. 204), the North American philosopher of art, Nöel Carroll, also: “treats film music more as supplement than complement since music for Carroll is one means, but not the only means, that film uses to clarify “the expressive quality” and “emotional significance of the action”.”

20 Ansani et al. (2020, p. 15) suggests that: “Another topic to explore is what it means that a music piece fits a visual scene; something similar has been proposed for audiovisual advertising (Herget et al., 2018), but one might deepen what are the effects provoked by music that does not fit a scene and whether this can elicit cognitive dissonance.” Note also that this point relates to the possibility of perceiving similarity across the senses (see Di Stefano and Spence, 2023).

21 It is worth noting here that the predictive processing framework has been recently applied to the study of cinema (e.g., Coëgnarts, 2025; Miller et al., 2024).

22 Here, one might be reminded of Meyer’s (1956, p. 31) influential work linking emotional response to music with the fulfilment or violation of expectations: “[m]ental activity tends to become conscious when reflection and deliberation are involved in the completion of the response pattern, that is, when automatic behaviour is disturbed because a tendency has been inhibited.”

23 Of course, here one might also draw attention to the likely difference in screen size (Troscianko et al., 2012), and the question of whether watching a film is undertaken as a solo (laboratory study) or group activity (e.g., in the cinema; see Williams et al., 2016).

24 Ehrenfels concluded that a melody is “something novel in relation to this sum, something that certainly goes hand in hand with but is distinguishable from the sum of elements…” (Ehrenfels, 1988, p. 83). A melody is a relational structure. Ehrenfels called ‘gestalt quality’ this relational structure amongst elements that transcends the elements themselves.

25 In a similar, albeit less problematic vein (given that aliens do not have feelings), one might consider the sound of the thereamin, which has nowadays become associated in the mind of cinema-goers with the presence of aliens (e.g., Wierzbicki, 2002; see also Murphy, 2006; Schmidt, 2010). Notice how congruency, in this case, would appear to be determined by nothing more than associative learning (Spence and Zhang, n.d.).
